# Revealing Volatile Odor Compounds in Watermelon Juice to Enhance Fructose Sweetness Perception: Sensory Evaluation and Molecular Docking Techniques

**DOI:** 10.3390/foods14061034

**Published:** 2025-03-18

**Authors:** Yixin Dai, Shuang Sun, Fan Yang, Shaobo Zhen, Xiaoying Xiong, Ye Liu, Shuang Bi

**Affiliations:** 1 Beijing Engineering and Technology Research Center of Food Additives, Beijing Advanced Innovation Center for Food Nutrition and Human Health, School of Food and Health, Beijing Technology and Business University (BTBU), No. 11, Fucheng Road, Haidian District, Beijing 100048, China; 2 School of Hotel Management, China University of Labor Relations, Beijing 100089, China

**Keywords:** odor-induced sweetness enhancement, watermelon juice, volatile odor compounds, sensory evaluation, molecular docking

## Abstract

Odor-induced sweetness enhancement (OISE) is an effective approach to lower sugar intake. In this study, static and dynamic sensory evaluations, combined with molecular docking, were used to explore the mechanism underlying sweetness enhancement in fructose solutions induced by watermelon juice odor compounds. Sensory evaluation results showed that the seven volatile odor compounds (VOCs) (ethyl acetate, ethyl propionate, octanal, (E,E)-2,4-hexadienal, (E)-2-octenal, methyl heptenone, and geranyl acetone) from watermelon juice could significantly increase the sweetness intensity of a 2.5% fructose solution, and the potency of OISE was significantly enhanced within 10 s. (E,E)-2,4-hexadienal, ethyl propionate, and methylheptenone showed the most significant OISE effects, which aligned with the sensory preference results. Further, molecular docking was used to explore the interactions between VOCs, fructose, and sweet receptors. The results showed that T1R2 was the main receptor for binding fructose and VOCs, and the interaction forces were primarily hydrogen bonds and hydrophobic interactions. In the presence of VOCs, the amino acid residues that formed hydrogen bonds with fructose were highly repetitive, with the main difference being the bond length, indicating the important role of flavor–sweetener receptor interactions in lowering fructose content and enhancing sweetness perception. Thus, this study provides a scientific basis for developing sugar-reduction technology based on aroma, in addition to enhancing sweetness.

## 1. Introduction

Sugar is an essential ingredient in the food industry, imparting various beneficial properties to food, such as acting as a preservative, stabilizer, dispersant, flavor precursor, and carrier [1]. However, the global per capita sugar intake is about 500 kcal per day, which is significantly higher than that recommended by the World Health Organization (25 g or 96 kcal) [2]. Excessive sugar consumption leads to overnutrition and chronic diseases, especially obesity, diabetes, and metabolic disorders [3,4]. Therefore, it is a crucial goal for the food industry to reduce sugar content while maintaining the quality and flavor of food.

Currently, sweeteners are mainly used to replace natural sugar sources to reduce sugar content in food production [3]. However, the safety of long-term consumption of sweeteners is controversial. For example, even low concentrations of sodium cyclamate (0.06 μM) can cause the microfilaments and microtubules of osteoblasts to curl and fold, and their vitality decreases significantly at higher dosages [5]. Erythritol increases the risk of cardiovascular diseases and adversely affects the liver [6,7]. In addition, there is a significant difference between the taste of sweeteners and natural sugar, making it difficult to meet consumer demand for food flavor [8]. In this context, cross-modal sensory interaction has gained widespread attention as an effective method to reduce sugar intake.

Food flavor perception involves complex cross-modal interactions among odor, taste, and chemical sensation [9]. As a phenomenon, odor-induced sweetness enhancement (OISE) refers to the presence of certain odors, resulting in an enhanced intensity of perceived sweetness. For example, the addition of 0.2% vanilla aroma to low-sugar yogurt effectively enhanced its sweetness perception and reduced the sugar content by 25% without compromising its quality and acceptability [10]. At the same time, vanilla aroma can enhance the sweetness intensity of different sweeteners [11]. Previous studies have found that ethyl butyrate, methyl 2-methylbutyrate, ethyl 2-methylbutyrate, and linalool significantly enhanced the sweetness of juice [12]. In addition, some green and aldehydic compounds enhance the sweetness perception of sucrose, such as β-pinene, tetradecanal, and dodecanol [13]. Similarly, fruity compounds in sweet orange, ethyl propionate, octyl acetate, and acetophenone significantly enhanced the sweetness intensity of the 5% sucrose solution [14]. Mango aroma also demonstrated OISE in low-concentration sucrose solutions, and a combination of 0.25% mango aroma and 2.24% sucrose produced the most significant OISE effect, with sweetness intensity comparable to that of 2.96% sucrose [3]. Additionally, aldehydes and esters with fruity aromas enhanced the sweetness perception of a sucrose solution [15].

Watermelon juice is popular among consumers due to its refreshing flavor and various health benefits [16]. In recent years, watermelon flavors have been widely used in new food products globally, including beverages, candy, and chewing gum. Therefore, watermelon’s natural odor compounds may be more acceptable to consumers for sugar reduction. Sweet, fruity, floral, and green aromas are the characteristic aromas attributes of watermelon juice, resulting from the abundance of alcohols, aldehydes, ketones, and esters in watermelon juice [16,17]. In flavor chemistry studies, the odor activity value (OAV) has become the gold standard for screening key odor molecules by comparing the ratio of a compound’s concentration to its olfactory threshold (OAV = C/OT) [18]. For example, recent studies on flat peach and apple have shown that OAV screening is effective in identifying volatile odor compounds (VOCs) that contribute substantially to the overall flavor [19,20]. However, the OISE effect of watermelon odor has not been explored. In our previous study, seven odor compounds with sweet aroma descriptions higher than 60% were identified in watermelon juice, including ethyl acetate, ethyl propionate, octanal, (E,E)-2,4-hexadienal, (E)-2-octenal, methylheptenone, and geranylacetone [16]. In addition, ethyl propionate and (Z)-6-nonenal have been proven to show OISE in a previous study [14]. Thus, it is reasonable to speculate that the odor compounds in watermelon juice may exhibit OISE, making it valuable for applications in sweetening and reducing the sugar content of foods.

Therefore, the purpose of this study was (i) to evaluate the sweetness perception induced by odor compounds from watermelon juice in fructose solution in the oral cavity using time-intensity (TI) and dynamic quantitative descriptive analysis (D-QDA); (ii) to assess the effects of different odor compounds on the acceptability of fructose solutions through sensory evaluation; (iii) to investigate the potential mechanism of sweetness induced by watermelon odor compounds via molecular docking. The findings of this study provide useful information for cross-modal interaction perception and offer new ideas for the development of OISE. This study also provides a theoretical basis for applying the odor compounds of watermelon juice to low-sugar beverages through cross-modal interaction to achieve the effect of sweetness while reducing sugar content.

## 2. Materials and Methods

### 2.1. Materials

Qilin variety watermelon was purchased from Yonghui Supermarket (Beijing, China) and processed into watermelon juice according to the method mentioned in a previous study [17].

Ethyl acetate, ethyl propionate, octanal, (E,E)-2,4-hexadienal, (E)-2-octenal, methylheptenone, and geranyl acetone were obtained from Quanpinsu Biotechnology Co., Ltd. (Beijing, China). Fructose was purchased from Wanbang Co., Ltd. (Henan, China). The water used in the experiments was Wahaha pure water, purchased from Wahaha Electronic Commerce Co., Ltd. (Hangzhou, China). All materials used in the experiments were of food-grade quality.

### 2.2. Determination of Concentrations of Sweet VOCs in Watermelon Juice

Selective ion monitoring (SIM) mode was used to quantify “sweet” VOCs in watermelon juice, using 2-methyl-3-heptanone (81.6 μg/L) as the internal standard added to each diluted standard solution, as described in a previous study [17]. The peak area ratio (Ax/Ai) and the concentration ratio (Cx/Ci) of each sweet odor compound to the internal standard were used to generate standard curves (Table S1).

### 2.3. Determination of Odor Activity Values (OAVs)

The OAVs were calculated as the ratio of the concentration of each “sweet” VOC to its respective odor threshold (OT) in a 2.5% fructose solution.

### 2.4. Sensory Evaluation Team

Referring to the previous screening method for sensory evaluation panelists [21], forty-six volunteers (29 females and 17 males) were recruited to evaluate preferences for fructose solutions with added odor compounds. Panelists had more than one year of experience in sensory evaluation. Details of the experiment were explained to sensory panelists prior to sensory testing and training, and all panelists agreed to participate in this study prior to participation. Sensory panelists were subjected to matching, triangulation, and sequencing tests. In the matching test, two of the five flavor solutions representing sour (citric acid, 5 mM), sweet (fructose, 10 mM), bitter (l-isoleucine, 1 mM), salty (NaCl, 12 mM), and fresh (MSG, 8 mM) flavors were randomly poured into tasting cups. Testers were asked to taste and determine the flavor attributes of the samples. In the triangulation experiment, a fructose solution and two aqueous solutions were prepared. Testers were asked to select the fructose solution from the three samples. In the sequencing experiment, testers were asked to sensory test five different concentrations of fructose solutions and rank them according to sweetness intensity from weakest to strongest. Of the three tests, 12 panelists (6 females, 6 males) with correct scores above 80% were selected for the QDA test of sweetness intensity of fructose solutions and underwent a one-month sweetness sensory training (Standard No. ISO 8589:2007) [22]. All evaluators provided informed consent before participation.

### 2.5. Determination of VOCs Thresholds in Fructose Solution

A simulated system was prepared by adding 2n stepwise dilutions of odor compounds to a 2.5% fructose solution. The thresholds of odor compounds in the fructose solutions were determined using the 3-alternative forced choice (3-AFC) method according to GB/T 22366-2022 [23]. The corrected detection probability was calculated using the following formula: p* = (3*p* − 1)/2, where p* denotes the corrected detection probability, and *p* denotes the actual detection probability. Using the S-curve method, data were plotted with the concentration of odor compounds at *p* = 0.5 representing the threshold [14].

### 2.6. TI Evaluation for Sweetness Intensity of Fructose Solutions Enhanced by Selected VOCs

The sweetness intensity of fructose solutions with added odor compounds was sensorially evaluated using the TI method [24]. Odor compounds were added to a 2.5% fructose solution at the determined concentrations to prepare different odor compound–fructose composite systems. Before the odor-induced sweetness-enhanced postnasal action, panelists wore nose clips before the evaluation to avoid cross-contamination from sniffing. After starting the timer, panelists sipped the solution from coded cups while removing the nose clip and instantly evaluated the sweetness intensity. The perceived sweetness of the samples was scored on a continuous line scale, with the intensity ranging from 0 to 9 (0 for no sweetness and 9 for intense sweetness). The reference sample contained a medium fructose concentration (2.5% *w*/*w*), corresponding to a sweetness score of 5. Each panelist evaluated the sample for 10 s and then spat it out immediately. The sensory panelists continued to assess the aftertaste until the sweetness disappeared. The total evaluation time lasted 50 s, and each panelist performed this process in triplicate, with a 3 min resting period between each sample to avoid olfactory fatigue and residual effects.

### 2.7. Sensory Evaluation of Sweetness Intensity of Fructose Solutions Enhanced by Selected VOCs

The sensory evaluation of odor-induced sweetness enhancement of fructose solutions was conducted using quantitative descriptive analysis (QDA) [24]. The test sample was prepared by adding VOCs to a 2.5% fructose solution. Before the formal experiment, panelists were asked to wear nose clips to obtain the postnasal olfactory and sweetness perception data. At the beginning of the evaluation, panelists removed the nose clip, swirled each sample in the mouth for 10 s, spat it out, and then scored the sweetness intensity of the system. Panelists rinsed their mouths with pure water between samples and rested for 3 min to avoid olfactory fatigue and residual effects.

### 2.8. Preference Evaluation of Fructose Solution with Selected VOCs

Forty-six trained sensory evaluators rated the preference for fructose solutions with added VOCs based on real feelings of olfactory, gustatory, and psychological aspects [3]. Panelists were asked to swirl 2 mL of the VOC–fructose mixture in their mouths for 10 s, then spit it out and rate the overall preference for the sample on a 9-point hedonic scale (1 for strongly dislike; 9 for strongly like). A 2.5% fructose solution was used as a reference for preference, corresponding to the median score of 5 points. Each panelist repeated this process thrice, with a 3 min rest between samples to avoid olfactory fatigue and residual effects.

### 2.9. Molecular Docking

The mechanism of interaction of fructose and seven sweet VOCs with sweet receptors was analyzed via molecular docking. The amino acid sequences of the TAS1R2 (T1R2) and TAS1R3 (T1R3) sweet receptor proteins were obtained from the UniProt database (https://www.uniprot.org, accessed on 3 January 2025), and the structure of sweet receptor dimer was predicted using AlphaFold 3.0. The SDF format file of VOCs was retrieved from the PubChem database and converted to PDB format using the Open Babel 3.1.1 software suite.

Molecular docking of VOCs with sweet receptors was performed using AutoDock Vina 1.2.5 (The Scripps Research Institute, La Jolla, CA, USA) [25,26]. Subsequently, D-fructose was docked to the VOCs-T1R2/T1R3 receptor protein model to study the changes in D-fructose after adding VOCs. Protein processing involves operations such as performing hydrogenation and charge addition, then calculating charges and adding atom types.

Next, conformationally optimized molecular docking was performed on the small molecules of VOCs, with the coordinates of the docking pockets set during the docking process predicted using DeepSite (https://www.playmolecule.com, accessed on 3 January 2025), which showed that the coordinates of the active center were (16.53, 20.1, −32.29). The Gird box was set to X: 26, Y: 26, and Z: 26. After 50 dockings, the best conformations were selected and saved as a PDB file, and the three-dimensional (3-D) structure was plotted using PyMol 3.0.0 (DeLano Scientific LLC, South San Francisco, CA, USA), and the two-dimensional structure was plotted using LigPlot+ 2.8.8 (European Bioinformatics Institute, Cambridge, UK).

### 2.10. Statistical Analysis

OriginPro 2019b (OriginLab Corp., Northampton, MA, USA) and Microsoft Excel 2019 (Microsoft Corp., Redmond, WA, USA) were used to generate images and tables. IBM SPSS Statistics 26 (International Business Machines Corp., New York, NY, USA) was used to conduct a one-way analysis of variance (ANOVA), Duncan’s multivariate test, and Pearson correlation analysis. Differences were considered statistically significant when Sig ≤ 0.05 or *p* ≤ 0.05.

## 3. Results and Discussion

### 3.1. Threshold and OAV of Sweet VOCs in Fructose Solution

Several previous studies have used the threshold in water to calculate OAVs to explore the contribution of odor compounds to the aroma of samples. In a previous study, a fructose solution was found to significantly affect the release of odor compounds [27]. Therefore, the threshold values of seven odor compounds in fructose solutions were determined using the S-curve method to ensure accurate odor perception by sensory evaluators. To visualize threshold changes, the ratio of the threshold of the odor compounds in a 2.5% fructose solution to their threshold in water was calculated. A ratio greater than 1 indicates an increased threshold.

As seen in Table 1, the measured threshold of methylheptenone in the fructose solution was reduced (ratio of 0.93). Specifically, the threshold of methylheptenone decreased from 68 μg/L to 63.41 μg/L, and its odor in fructose solution was more prominent, which was described as “citrus, apple, and green”. The threshold of geranyl acetone in fructose solution was similar to that of water, contributing “fruity, floral, and green” aromas. Other odor compounds showed higher thresholds in fructose solution than in water. In particular, the octanal threshold increased from 0.8 μg/L to 4.27 μg/L, which had odor attributes such as “citrus and green”, and the multiple difference of its threshold was the largest (5.34). This increase in the threshold may be due to the interactions between fructose and VOCs, which inhibit the release of odor compounds. This is consistent with the results from previous studies, wherein hydrogen bonding, hydrophobic interactions, and van der Waals forces were observed between fructose and VOCs such as hexanal and nonanal, leading to a significant reduction in their release [27]. Moreover, fructose exhibits strong intermolecular polarity, resulting in reduced volatility of odor compounds [8].

The seven sweet VOCs were quantitatively analyzed using the standard curve method (Table 1). Among the seven odor compounds, the highest OAV was shown by (E,E)-2,4-hexadienal, which is also called “ muskmelon aldehyde”, and had “muskmelon, floral, and green” odor properties [16]. The OAVs of all seven odor compounds were greater than 1, indicating that the corresponding concentration of sweet VOCs in watermelon juice allowed the sensory evaluator to perceive their odor characteristics in fructose solution. Thus, the enhancement effect of VOCs on the sweetness intensity of a 2.5% fructose solution can be explored at these concentrations.

### 3.2. Dynamic Perception of Sweetness Intensity of Fructose Solution Induced by Sweet VOCs

**Figure 1:**
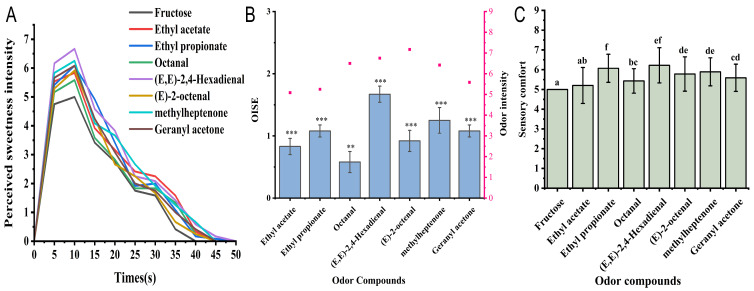
Sensory evaluation of sweetness intensity of fructose solution induced by VOCs in watermelon juice. (**A**) Dynamic evaluation of sweetness intensity changes of fructose induced by nasal odor by TI method; (**B**) OISE effect of VOCs. Stars represent significant differences from 0: *** *p* < 0.001; ** *p*  <  0.01 (**C**) Evaluation of overall preference of VOCs for fructose solution; different letters represent significant differences (*p* < 0.05). Error bars represent the standard errors of the mean.

The sweetness intensity of fructose solutions during oral processing was measured using the TI method, including the evaluation of sweetness aftertaste for 50 s. The dynamic sensory results of the sweetness intensity of a 2.5% fructose solution with added VOCs are shown in Figure 1A.

The sweetness perception of the fructose solution initially increased and then decreased. The highest sweetness intensity was perceived at 10 s; however, after 15 s, the sweetness perception intensity decreased significantly, with the duration of perception being 40 s. After the addition of seven VOCs, the highest sweetness perception time of the fructose solution was still 10 s. However, it significantly increased the sweetness intensity and slowed the decrease in the sweetness intensity sensory scores. The duration of sweetness perception induced by VOCs was prolonged to more than 45 s, with ethyl propionate and (E,E)-2,4-hexadienal extending the perception time of fructose solutions to 50 s. This result shows that “sweet” VOCs can significantly increase the sweetness intensity and prolong the sweetness perception time of fructose solutions. This is consistent with the research showing that pyrazine compounds can prolong the salty taste duration of a 0.2% NaCl solution [24].

### 3.3. Evaluation of Sweetness Enhancement on Fructose Solutions by Sweet VOCs

The sweetness enhancement effect of VOCs on fructose solutions was assessed by calculating the OISE. As shown in Figure 1B, the baseline (0) is the sweetness intensity of a 2.5% fructose solution.

Ethyl acetate, ethyl propionate, octanal, (E,E)-2,4-hexadienal, (E)-2-octenal, methylheptenone, and geranyl acetone significantly increased the sweetness intensity of 2.5% fructose solutions. Among them, (E,E)-2,4-hexadienal, with a “melon, fruity, and green” flavor, showed the most significant sweetness enhancement effect (*p* < 0.001), with its OISE reaching 1.67. The OISE of ethyl propionate in fructose solution was 1.08, and it has been reported to increase the sweetness intensity of 5% sucrose solution at low concentrations [14]. The OISE of ethyl acetate, described as “fruity, green, and sweet”, was 0.83 (*p* < 0.001) in the 2.5% fructose solution, while, in another study, ethyl acetate did not show significant sweetness enhancement of 30 g/L sucrose solution [28], possibly due to the addition of a different concentration of ethyl acetate. Additionally, the sweetening effect depends on the type and concentration of the odor compound [12], and the type of sugar used, which can explain the difference in OISE results.

### 3.4. Evaluation of Sensory Preference of Fructose Solutions After Adding Sweet VOCs

Sensory preference is an important indicator of consumers’ choice and repurchase of food products [29]. Sensory evaluators assessed the overall preference for fructose solutions with different VOCs using a nine-point rapture scale, as shown in Figure 1C. All seven VOCs significantly increased the overall preference for fructose solutions (*p* < 0.05). Among them, (E,E)-2,4-hexadienal, ethyl propionate, and methylheptenone had the highest preference scores, which were 6.22, 6.07, and 5.89, respectively, indicating that the sensory preference was consistent with their sweetness intensity. “Fruity” aroma compounds are associated with positive emotions, such as happiness and relaxation, in consumers [30]. This is consistent with the research results of Xiao et al., where the sensory comfort of sucrose solution was improved by the addition of ethyl propionate [14].

### 3.5. Binary Interaction Between Fructose and T1R2/T1R3

**Figure 2:**
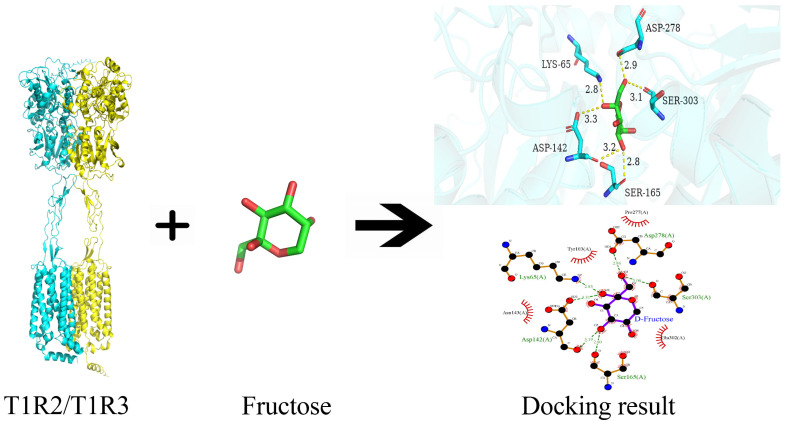
Binary interaction between fructose and sweet taste receptor T1R2/T1R3.

Figure 2 depicts the interaction of the sweet taste receptors T1R2/T1R3 dimer with fructose. The minimum binding energy between fructose and T1R2/T1R3 was −5.7 kcal/mol, indicating a relatively stable binding affinity [31]. The hydroxyl groups of fructose formed hydrogen bonds with the amino groups in the amino acid side chains of T1R2/T1R3, primarily with Asp278, Ser303, Ser165, Asp142, and Lys65, with bond lengths of 2.86, 3.06, 2.80, 3.19, and 3.31, and 2.85, respectively. In addition, fructose exhibited hydrophobic interactions with Pro277, Glu302, Asn143, and Tyr103. Molecular docking results showed that the fructose interacted with the sweet taste receptors with the Venus flytrap domain (VFD) of T1R2, which was consistent with the results obtained by Mahalapbutr [32] and Niu et al. [8], suggesting that the VFD on the T1R2 monomer was the preferred binding site for certain sweeteners.

### 3.6. Ternary Interaction of VOCs, Fructose, and T1R2/T1R3

**Figure 3:**
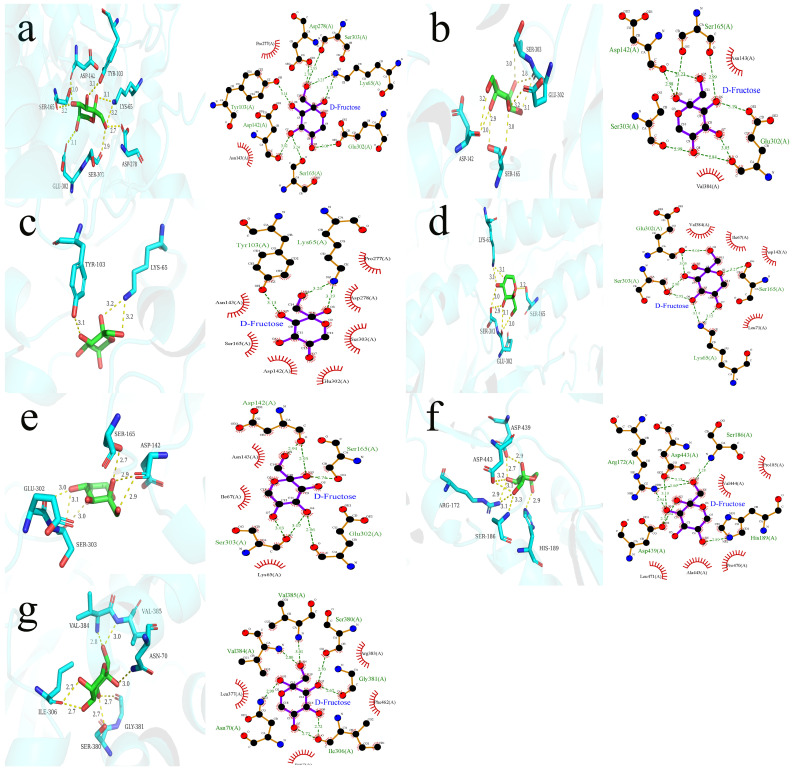
Ternary interaction of fructose–VOCs–T1R2/T1R3. (**a**) Ethyl acetate; (**b**) ethyl propionate; (**c**) octanal; (**d**) (E,E)-2,4-hexadienal; (**e**) (E)-2-octenal; (**f**) methylheptenone; (**g**) geranyl acetone.

First, the interaction between VOCs and sweet taste receptors formed a binary complex, followed by docking with fructose to explore the influence of VOCs on the sweetness perception of fructose solutions. As shown in Figure 3, the interaction region for VOCs, fructose, and T1R2/T1R3 was within the VFD of T1R2. As shown in Table 2, the binding energies of fructose to T1R2/T1R3 in the presence of different VOCs ranged from −5.77 to −5.282 kcal/mol, and, the stronger the binding force of the receptor, the lower the binding energy. In particular, the complexes of ethyl acetate, ethyl propionate, and (E,E)-2,4-hexadienal–fructose with T1R2/T1R3 showed stronger affinities, with binding energies of −5.77, −5.563, and −5.711 kcal/mol, respectively. In contrast, the ketones (methylheptenone and geranyl acetone) displayed higher binding energies of −5.282 and −5.38, respectively. Sensory evaluation showed that (E,E)-2,4-hexadienal and methylheptenone demonstrated the most significant OISE in fructose solutions; however, molecular docking indicated that their binding energies were not the lowest. This inconsistency between binding energy and sensory evaluation results may be due to some deviation between the T1R2/T1R3 homology model and the real receptor structure [33]. Moreover, the mechanism of OISE may involve associative learning, natural association, and positive allosteric modulators (PAM) of the sweet taste receptors [34], and is the result of a combination of psychology, neurology, and sensory science [35].

As shown in Table 2 and Figure 3, the main forces involved in the ternary interaction among T1R2/T1R3–VOCs–fructose were hydrogen bonding and hydrophobic interactions. Structurally, the oxygen-containing groups in fructose facilitated the interactions with amino acid residues (amines and carboxyl groups) to form hydrogen bonds [8]. The main amino acid residues that formed hydrogen bonds between the ligands and the sweet taste receptors included Ser303, Ser165, and Lys65. In the presence of ethyl acetate, the residues forming hydrogen bonds, such as Asp278, Ser303, Ser165, Asp142, and Lys65, were the same as those found in the fructose–T1R2/T1R3 interaction. In the presence of ethyl acetate, octanal, and (E,E)-2,4-hexanedial, the formation of hydrogen bonds involved the residue Lsy65. In contrast, the lower binding energies of fructose and sweet taste receptors in the presence of ethyl propionate, (E)-2-octenal, methyl heptenone, and geranyl acetone were mainly distinguished by the formation of hydrogen bonds without the involvement of residue Lys65. This suggests that there is a similar pattern of fructose–sweetness receptor interactions in the presence of the three compounds, ethyl acetate, octanal, and (E,E)-2,4-hexanedial, and that the Lys65 residue plays a more critical role in these interactions, which affects the recognition of fructose [36]. The hydrophobic interactions between ligands and sweet taste receptors mainly included amino acid residues, such as Pro277, Ala445, Ser303, and Tyr103. Notably, Tyr103 was also a key residue in the interaction between sucrose and sweet taste receptors [37]. (E)-2-octenal and ethyl propionate interacted with the same hydrogen-bonding residues but with distinctly different hydrophobic residues. The hydrophobic residues of (E)-2-octenal were Asn143, Lys65, and Ile67. The hydrophobic residues of ethyl propionate were Val384 and Asn143. Thus, these residues enhanced the hydrophobic interactions between fructose and sweet taste receptors, improving the stability of their interactions and further intensifying the sweetness perception of the fructose solution.

## 4. Conclusions

In the present study, ethyl acetate, ethyl propionate, octanal, (E,E)-2,4-hexadienal, (E)-2-octenal, methylheptenone, and geranyl acetone could significantly increase the sweetness intensity of fructose solutions and slowed the decline in sweetness intensity perception, extending the fructose sweetness perception duration to more than 45 s. Among them, (E,E)-2,4-hexadienal, ethyl propionate, and methylheptenone significantly increased the preference for fructose, indicating a consistency between the overall preference and sweetness intensity. The binding energies of fructose with T1R2/T1R3 ranged from −5.61 to −5.282 in the presence of different VOCs. The main forces involved in the ternary interaction of T1R2/T1R3–VOCs–fructose were hydrogen bonding and hydrophobic interactions. The main amino acid residues, such as Ser106, Glu302, and Asp14, formed hydrogen bonds between ligands and sweet taste receptors, while key residues such as Pro277, Ala445, Ser303, and Tyr103 contributed to the hydrophobic interactions. Thus, in the presence of VOCs, the amino acid residues involved in the formation of hydrogen bonds were highly repetitive, with the main difference being the bond length. These findings elucidate the sweetening effects and mechanisms of sweet VOCs in watermelon juice on fructose solutions, providing new insights into the application of watermelon odor compounds in low-sugar products to enhance their sweetness perception. To further promote practical applications in the future, the synergistic stability of VOCs can be evaluated in complex food systems by modeling the kinetics of pH, temperature, and flavor release.

## Figures and Tables

**Table 1:** Concentration of sweet aroma compounds in watermelon juice; threshold in fructose solution and OAV.

No.	Compounds	Odor Description	Threshold in Water (μg/L)	Threshold in 2.5% Fructose Solution (μg/L)	Ratio (Fructose Solution: Water)	Concentration in Watermelon Juice (μg/L)	OAV
1	Ethyl acetate	Fruity, green, sweety	5	11.57	2.31	112.35	10
2	Ethyl propionate	Fruity, sweety	10	12.36	1.24	14.57	1
3	Octanal	Citrus, green	0.8	4.27	5.34	32.91	8
4	(*E*,*E*)-2,4-hexadienal	Muskmelon, floral, green	1.8	5.08	2.82	124.35	24
5	(*E*)-2-octenal	Citrus, green	2.7	4.11	1.52	55.46	14
6	Methylheptenone	Citrus, apple, green	68	63.41	0.93	111.67	2
7	Geranyl acetone	Fruity, floral, green	60	63.42	1.06	65.85	1

**Table 2:** Binding energy, interaction forces, and key amino acid residues in the docking of T1R2/T1R3–fructose and T1R2/T1R3–aroma compound–fructose combinations.

Combination	Binding Energy (kcal/mol)	Hydrogen Bonding Residue Amino Acid	Hydrophobic Residue Amino Acid
T1R2/T1R3–fructose	−5.7	Asp278, Ser303, Ser165, Asp142, Lys65	Pro277, Glu302, Asn143, Tyr103
T1R2/T1R3–ethyl acetate–fructose	−5.77	Asp278, Ser303, Ser165, Asp142, Lys65, Tyr103, Glu302	Pro277, Asn143
T1R2/T1R3–ethyl propionate–fructose	−5.563	Ser303, Glu302, Ser165, Asp142	Val384, Asn143
T1R2/T1R3–octanal–fructose	−5.714	Tyr103, Lys65	Pro277, Ser303, Asn143, Asp278, Ser165, Asp142, Glu302
T1R2/T1R3-(*E*,*E*)–2,4-hexadienal–fructose	−5.711	Lsy65, Ser303, Glu302, Ser165	Val384, Leu71, Asp142, Ile67
T1R2/T1R3–(*E*)-2-octenal–fructose	−5.625	Asp142, Ser165, Glu302, Ser303	Asn143, Lys65, Ile67
T1R2/T1R3–methyl heptenone–fructose	−5.282	Asp439, Arg172, Ser186, Asp443, His189	Val444, Ala445, Leu471, Pro470, Pro185
T1R2/T1R3–geranyl acetone–fructose	−5.38	Asn70, Val384, Val385, Gly381, Ser380, Il306	Arg383, Ile67, Phe462, Leu377

## Data Availability

The original contributions presented in this study are included in this article/Supplementary Materials. Further inquiries can be directed to the corresponding authors.

## Supplementary Materials

The following supporting information can be downloaded at: https://www.mdpi.com/article/10.3390/foods14061034/s1, Table S1. The quantification of the VOCs of watermelon juice; Figure S1. Threshold values of VOCs in 2.5% fructose solution.

## Abbreviations

The following abbreviations are used in this manuscript:

OISE	Odor-induced sweetness enhancement
VOCs	Volatile odor compounds
TI	Time-intensity
LD	Dynamic quantitative descriptive analysis
SIM	Selective ion monitoring
OT	odor threshold
VFD	Venus flytrap domain
