# Supplementary material for: Revealing Volatile Odor Compounds in Watermelon Juice to Enhance Fructose Sweetness Perception: Sensory Evaluation and Molecular Docking Techniques
Source: Foods. 2025 Mar 18;14(6):1034. doi: 10.3390/foods14061034 (PMC11942085; doi:10.3390/foods14061034)
Supplement: Supplementary file 1 [file foods-14-01034-s001.zip › foods-3522077-supplementary.pdf]

Supplementary Material

Table S1. The quantification of the VOCs of watermelon juice

No.	Compounds [a]	Linear equations [b]	R^2	Concentration range (μg/L) [c]	Quota selected ion [d]
1	Ethyl acetate	y = 0.5289x + 0.0266	0.9933	18-450	43, 61, 88
2	Ethyl propionate	y = 0.4982x + 0.0064	0.9976	18-450	57, 74, 102
3	Octanal	y = 0.4528x + 0.0102	0.9984	16-450	56, 84, 110
4	*(E,E)*-2,4-Hexadienal	y = 0.4613x + 0.0407	0.9902	18-450	67, 81, 96
5	*(E)*-2-octenal	y = 0.0434x + 0.0009	0.9932	16-400	55, 70, 83
6	Methylheptenone	y = 0.5423x + 0.0195	0.9934	16-400	55, 69, 108
7	Geranyl acetone	y = 0.4809x + 0.0213	0.9915	17.46-436.5	43, 69, 194

a Sweety compounds in watermelon juice.

b Linear equations were fitted by the peak area and corresponding concentration in watermelon juice.

c Ranges of each compound concentration for provided linearity.

d Ions selected for quantitative analysis.

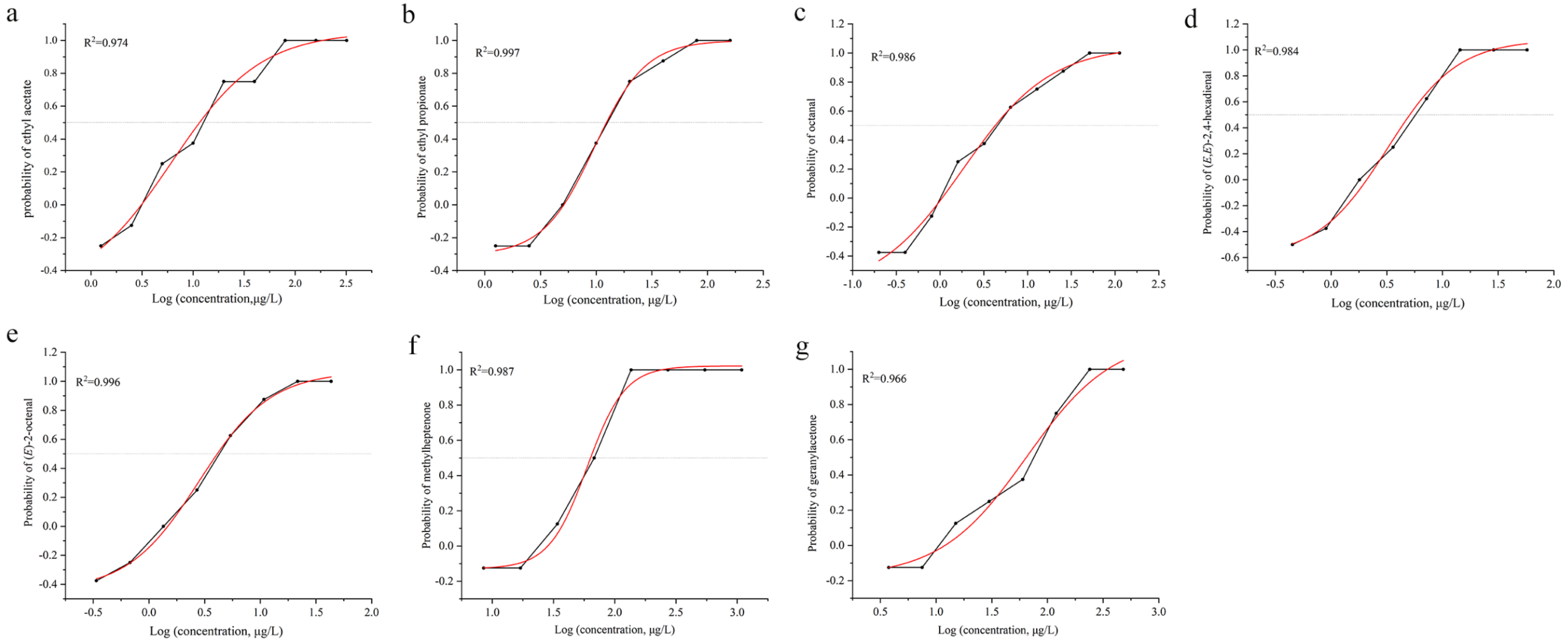

Fig. S1 Threshold values of ethyl acetate (a), ethyl propionate (b), octanal (c), (*E*,*E*)-2,4-hexadienal (d), (*E*)-2-octenal (e), methylheptenone (f) and geranyl acetone (g) in 2.5 % fructose solution; Black square: raw data; Red line: Threshold fitting curve.
